# Assessing the context within academic health institutions toward improving equity-based, community and patient-engaged research

**DOI:** 10.1017/cts.2024.675

**Published:** 2024-12-11

**Authors:** Prajakta Adsul, Shannon Sanchez-Youngman, Elizabeth Dickson, Belkis Jacquez, Alena Kuhlemeier, Michael Muhammad, Katherine J. Briant, Bridgette Hempstead, Jason A. Mendoza, Lisa G. Rosas, Anisha Patel, Patricia Rodriguez Espinosa, Tabia Akintobi, Paige Castro-Reyes, Lori Carter-Edwards, Nina Wallerstein

**Affiliations:** 1 Department of Internal Medicine, School of Medicine, University of New Mexico Health Sciences Center, Albuquerque, NM, USA; 2 Cancer Control and Population Sciences Research Program, Comprehensive Cancer Center, University of New Mexico, Albuquerque, NM, USA; 3 College of Population Health, University of New Mexico, Albuquerque, NM, USA; 4 Center for Participatory Research, University of New Mexico, Albuquerque, NM, USA; 5 Community Outreach and Engagement Office, Fred Hutchinson Cancer Center, Seattle, WA, USA; 6 Cierra Sisters Inc., Seattle, WA, USA; 7 Department of Epidemiology and Population Health, Office of Community Engagement, Stanford University School of Medicine, Palo Alto, CA, USA; 8 Department of Pediatrics, Stanford, Palo Alto, CA, USA; 9 Morehouse School of Medicine, Atlanta, GA, USA; 10 Community-Campus Partnerships for Health, Raleigh, NC, USA; 11 Kaiser Permanente Bernard J. Tyson School of Medicine, Pasadena, CA, USA

**Keywords:** Patient and community engagement, community-based participatory research, academic health institutions, institutional context, implementation science

## Abstract

**Introduction:**

The continued momentum toward equity-based, patient/community-engaged research (P/CenR) is pushing health sciences to embrace principles of community-based participatory research. Much of this progress has hinged on individual patient/community–academic partnered research projects and partnerships with minimal institutional support from their academic health institutions.

**Methods:**

We partnered with three academic health institutions and used mixed methods (i.e., institution-wide survey (*n* = 99); qualitative interviews with institutional leadership (*n* = 11); and focus group discussions (6 focus groups with patients and community members (*n* = 22); and researchers and research staff (*n* = 9)) to gain a deeper understanding of the institutional context.

**Results:**

Five key themes emerged that were supported by quantitative data. First, the global pandemic and national events highlighting social injustices sparked a focus on health equity in academic institutions; however, (theme 2) such a focus did not always translate to support for P/CenR nor align with institutional reputation. Only 52% of academics and 79% of community partners believed that the institution is acting on the commitment to health equity (Χ^2^ = 6.466, *p* < 0.05). Third, institutional structures created power imbalances and community mistrust which were identified as key barriers to P/CenR. Fourth, participants reported that institutional resources and investments are necessary for recruitment and retention of community-engaged researchers. Finally, despite challenges, participants were motivated to transform current paradigms of research and noted that accountability, communication, and training were key facilitators.

**Conclusions:**

Triangulating findings from this mixed-methods study revealed critical barriers which provide important targets for interventions to improving supportive policies and practices toward equity-based P/CenR.

## Introduction

Community-based participatory research (CBPR) and patient/community-engaged research (P/CEnR) projects have been established over the last two decades with integration of engagement principles [1,2], resulting in a growing body of evidence of the impact of this approach on social and health improvement outcomes [3–5]. CBPR is a collaborative research approach that actively involves community members and stakeholders in all stages of the research process to ensure that findings are relevant, applicable, and beneficial to the community itself [1,6]. Despite the integration of principles and recognition of positive outcomes, incorporation of promising or best collaborative practices remains fragmented and highly varied in research projects, with insufficient research support infrastructures and processes to help individual investigators and institutions create and sustain community–academic research partnerships [7–11]. The COVID pandemic re-ignited concern for inequities and racism, and with the murder of George Floyd, has strengthened the need to solidify investments in structural supports for community engagement [12–14].

A review of partnership engagement in Patient Centered Research Outcomes Research Institute (PCORI) funded projects highlights the importance of leveraging existing institutional infrastructures; and the importance of respecting and prioritizing the diversity of patient perspectives and values, especially from marginalized populations [15]. In a recent article by Carter-Edwards and colleagues, the authors note the lack of supportive institutional policies and procedures as well as fiscal and administrative processes that can foster P/CEnR [8]. This is supported by recent publications that stress the need for greater training and development of tools for patient and community engagement in research [16–18]. For national success, it is imperative for institutions to understand and enhance institutional internal capacity to support P/CEnR, internal and external structures needed, and institutional commitment to community and patient-centered health equity research with marginalized diverse populations to ensure empowerment through joint patient and community decision-making and shared governance in research.

To tackle these issues, the University of New Mexico Center for Participatory Research (UNM-CPR), with national partners, received a PCORI engagement award (2021–2023) which was built on three funding cycles from NIH Engage for Equity (E2) since 2006, from the UNM-CPR with national partners, producing a conceptual model for CBPR [6], and identifying partner best practices such as trust-building [19], culture-centeredness [20], power-sharing [21], formal agreements and other structures of co-governance [22], and collective empowerment [23], shown to contribute to outcomes 24,25].[Most recently, we conducted a randomized control trial of the E2 toolkit that strengthened the evidence for workshops versus website resources (available at: http://engageforequity.org) for strengthening partnership practices and outcomes [26].

While E2 has proven successful at supporting research projects at the partnership and individual level, E2 PLUS [27], described in this manuscript and funded by PCORI, sought to take the next step of scaling up the E2 for institutional transformation [27]. The UNM-CPR invited partners from three institutions for this project: Morehouse School of Medicine, Fred Hutchinson/University of Washington/Seattle’s Children’s Cancer Consortium, and Stanford School of Medicine and Cancer Institute. While details of the intervention are provided elsewhere [27,28], in brief, the E2 PLUS intervention consisted of establishing champion teams of investigators, staff, patient, and community advocates; collection and co-interpretation of quantitative and qualitative data about the institutional context of equity and engagement from top leaders, investigators, and patients and community through workshops; and bidirectional (i.e., between the UNM team and the champion team) coaching for the use of data and learnings to advocate for policy and practice changes to the top leaders at each participating institution. This paper provides a deeper understanding of institutional contexts, aggregated across the three participating institutions, as assessed by qualitative and quantitative methods from the perspectives of institutional leaders, investigators and community/patient members engaged within each institution.

## Materials and methods

Each site participated in the quantitative and qualitative institutional assessments; and for this analysis, we triangulated data[29] to generate a list of factors for within and across institutions that influenced the support toward and impact of P/CEnR. The study was reviewed and approved by University of New Mexico Health Sciences Center IRB (HRRC: # 21–320).

For the qualitative assessments, we conducted internet-based focus groups with 6–8 individuals at each site with two groups – one group consisting of researchers and research staff and the other group of patients, patient advisory committee members, community members, and community/patient advocates – for reflection on perceived issues regarding institutional support for P/CEnR and available institutional capacities. Since the groups were small and individuals would be easily identified, we did not collect any demographic information. A total of six focus groups (*n* = 22 patient or community members and *n* = 9 researchers and research staff) were conducted from the Fall 2021 to Summer of 2022. We also conducted three to four interviews of top leaders (e.g., Principal Investigators or Directors of Clinical Translational Science Awards Centers, Cancer Centers, or Prevention Research Centers, etc.) at each institution (*n* = 11) to assess their perspectives and vision for P/CenR promoting policies, practices, and resources and how these fit with their vision for equity. All group discussions were recorded and transcribed; transcripts were used for the analyses.

For the quantitative assessments, concurrent to the focus groups and interviews, champion teams recruited up to 35 individuals per site (total *n* = 99) (including other researchers/staff; outreach staff across the institutions; patients and community/patient advisory members; and selected leaders, such as training or IRB directors, and research or finance directors). Survey measures focused on institutional commitment to health equity, internal capacities, policies and processes, and external institutional influences related to P/CEnR, and is described elsewhere[30].

### Theoretical frameworks informing the analyses

**Figure 1.**
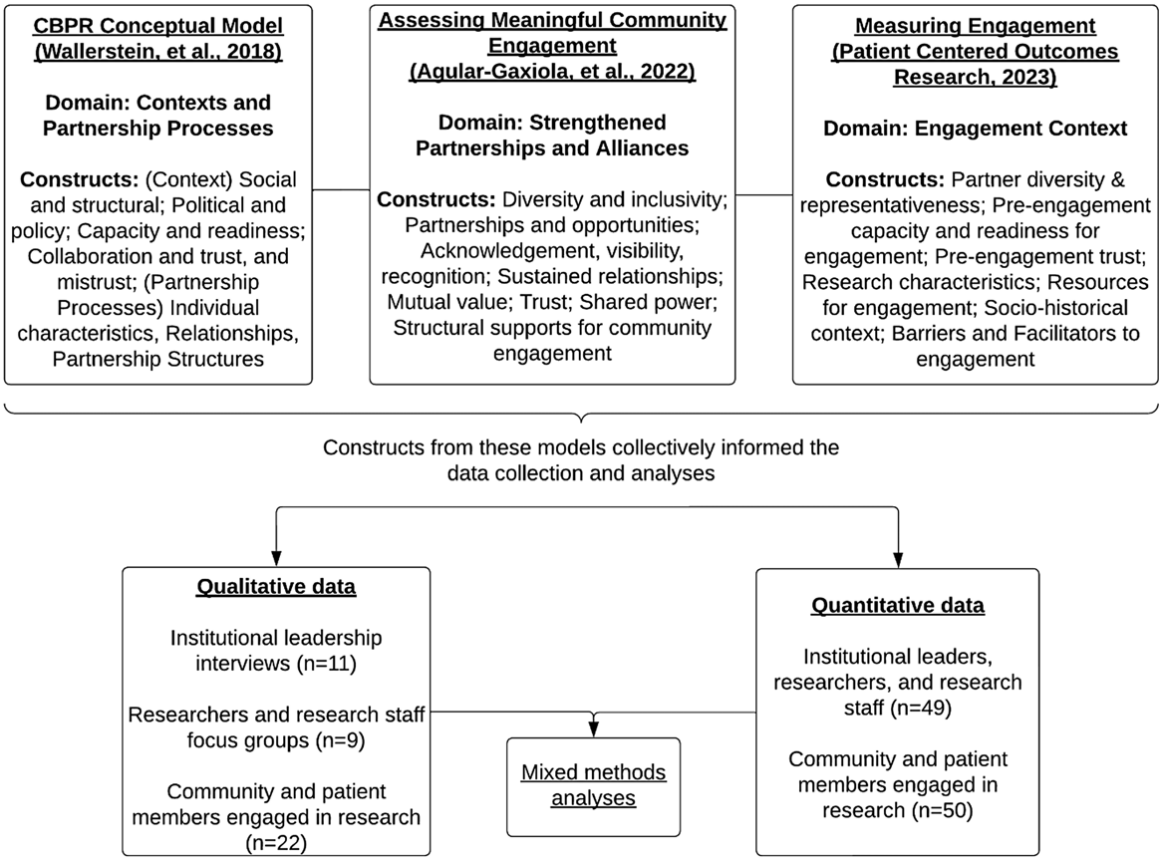
Theoretical frameworks, domains, and constructs, informing the analytical strategy.

The analytical strategy was guided by a comprehensive theoretical review [1,31,32]. First, we were guided by our own validated CBPR conceptual model that outlines the “context” under which community–academic partnerships operate [1,6]. This construct is often explored qualitatively, through the use of the collaboratively constructed “river of life” tool that helped workshop participants document their history of engagement across each institution, including facilitators and barriers they have faced [26,33]. A subconstruct under the context domain specifies the capacity of the academic partners or the institution, which was further validated in a study with community partnerships [6]. A goal of the present study was to explore and further develop our understanding of institutional context as it influences community-engaged research. Second, we also reviewed the newly developed Assessing Community Engagement (ACE) Conceptual Model [31], which reflects the major indicators leading to the fundamental goal of health equity and systems transformation while centering on community engagement. Although academic health institutions were not an explicit focus of this model, the domain of strengthened partnerships and alliances details key indicators such as sustained relationships, mutual value, trust, and structured supports for community engagement, which were key to consider. Another framework that influenced our analysis was the Engagement in Research: Theory of Action, by the PCORI and based on a landscape assessment conducted by RAND [34]. Similar to the ACE model, the PCORI Engagement in Research does not focus on academic health institutions; however, it does bring to focus the concept of context in which the engagement occurs, including the research setting and types of projects. Finally, we also informed our analysis by a review of the literature that highlighted key barriers at the institutional level[8] and from the perspectives of patients involved in research [18]. It is important to note that none of these frameworks were a clear fit for the proposed research question: What are important contextual influences in an academic health institutions that can support or hinder patient/community-engaged research? The domains and subconstructs within these frameworks that were often titled “context” or “partnerships,” however, critically informed the thematic analyses presented in the paper (see Figure 1 for specific domains and constructs).

### Analyses

We used a mixed-methods triangulation approach[29] to integrate findings from top leader interviews (*n* = 11) and discussions (*n* = 6 focus groups) with investigators and community/patient partners across the three academic health institutions, with surveys of respondents (*n* = 99) from all participating groups. First, we conducted analyses on the transcripts using an inductive and deductive process, informed by the theoretical review presented in Figure 1. All analyses were conducted in Atlas ti. Once the themes were outlined and described, we reviewed the quantitative reports to identify data points that either supported or opposed the qualitative findings. For themes that warranted comparisons of perspectives of community and academic partners separately, chi-square tests were performed on simplified versions of the variables of interest to assess whether differences between community and academic partners were significant. Simplified versions of the items had three categories (e.g., “Agree,” “Neither or Agree nor Disagree,” and “Disagree”) – collapsing variations in strength of agreement or disagreement and excluding “Don’t know” responses. Post hoc tests, using the Bonferroni method, were conducted to confirm significant differences between academic and community partners in the category reported. All quantitative analyses were conducted in R version 4.3.2. Additional details around the quantitative analyses are presented elsewhere [30]. In this manuscript, we focus only on results that informed the mixed-methods analyses, presented in a joint display in the results. Within the project, these data informed the ongoing reflection and strategic planning for the champion teams, through monthly meetings with UNM to reformulate goals, strategies, and actions as they relate to the CBPR model [35].

## Results

A total of 22 community members and patients and nine investigators or researchers participated in the focus group discussions, across the three institutions. We interviewed 11 institutional leaders (e.g., President and Director of an NCI-designated comprehensive cancer center, Chief Executive Officer of an academic health system, Directors of Clinical Translational Science Centers among others). Below, we present the key emergent themes and supporting quotes from the analyses in Tables 1, 2, and 3.

**Table 1.** Representative quotes for themes one and two

Theme 1: Global pandemic and national events highlighting social injustices sparked a focus on health equity in academic health institutions	
Q1	“And the disconnect isn’t just there on politics, it’s there in healthcare, it’s there in people’s inability to take the vaccine it’s people who think, the disinformation that’s out there is a real barrier for us to overcome. And understanding people who are different and why they are different is going to be one of the big goals… If we don’t keep our country together by recognizing that people of color have had a different experience in America and that their voice needs to be heard…health could be one of the things that brings [us together].” – Inst 1, Leader
Q2	“I think the pandemic is pulling some of the major activities that have historically been more separate, together, into that overall goal of accelerating what we’ve learned from discovery to improving the health of each individual patient and being able to measure the health of the population we’re studying” – Inst 1, Leader
Q3	“After George Floyd’s murder…a lot of leaders made pledges at that time. The pledge here said that “we will no longer be silent” I think there was an acknowledgement in the pledge that to date, we had been silent. They said, “we will use our influence to effect change,” speaking to our responsibility as a leading academic medical center to address issues related to racism as a public health crisis and they said “enough is enough,” echoing how we all felt and how we continue to feel about racial inequities and racial injustices.” – Inst 2, Leader
Q4	“We did this really cool thing…when telehealth happened, the only clinic that was really using telehealth prior to the pandemic was the type 1 diabetes, because they already recognized that with continuous glucose monitors that could be downloaded to the electronic medical record, you didn’t really need to bring the kid in, so they were already ahead of the curve on telehealth.” – Inst 2, Leader
Q5	“Some people like to think “oh, social justice started in June of 2020” not true. Meaning, there have been people who have been looking at these questions for decades, some people for hundreds of years, but for decades. And I think what we’re seeing now is that there is a broader understanding, at least among people at my institution.” – Inst 1, Leader
Q6	“There’s always ebbs and flows in research, where there may be a sexy topic of the day and they’re throwing money at it. We’re probably in such a phase right now with health equity, but we know that that is going to dry up like it usually does. And at least the institution has always been, I think, supportive in finding the resources to continue that kind of work and we take it to heart.” Inst 3, Leader
Q7	“Real shift that has happened, we are at a point now where the school is prioritizing. I had a meeting with the dean the other day and they really are prioritizing their fundraising in issues of health equity and access. It is new, it is a change, and I hope that we’ll see in the coming year, that moving forward we’re bringing in, we’re creating a real authentic funded pace for that …and that is not just window dressing.” Inst 2, Investigator
Q8	“I feel like there is a big shift now which is good, which is a real change from the time I was here in 2005 where health equity and community engagement and even CBPR in internal medicine wasn’t known, thought of or taught in our disciplines…But here is a big push and a discussion of these topics and I think there is a lot of discourse and connections as opposed to isolated groups that are working in these areas.” Inst 2, Investigator
**Theme 2: Including a focus on health equity did not translate to support for community-engaged research nor align with institutional reputation**	
Q9	“One of the interesting things, is what’s the difference between Diversity, Equity and Inclusion (DEI) and community outreach and engagement, I think that will be very interesting to see how that plays out. I think there is tremendous overlap there, which is fine, I don’t think that’s a problem. If that’s our biggest problem is overlap between those two areas, we’ve been successful.” – Inst 1, Leader
Q10	“I think CBPR [Community Based Participatory Research] is abused as a term…CBPR and health equity have become buzz words so we’re going to use them to talk about recruiting diverse populations. I have a hard time with precision health, how do you really talk about it at the same time, the initiative I’m working on is a research project. Trying to think about precision health at the level of the community. I just feel like the window dressing thing and the use and misuse of the language is still a big offense.” Inst 2, Investigator
Q11	“it could be a patient, it could be an organization, it could be the leaders of an organization, it could be local church leaders who might facilitate recruitment. We need to be able to have a more defined understanding, we need all of those community partners involved in most of our research, if not all of our research.” – Inst 2, Leader
Q12	“You’ll have to figure out what’s real [community engagement] …what is happening in the community, what are the barriers, and what prevents a woman from [area name] to get a mammogram…That, to me, is community informed research as opposed to doing a survey and then fitting in what you’re already doing and saying it’s responsive to the community. I don’t mean to be so cynical, but that’s most of what cancer centers are doing when they say they are meeting the needs of their catchment area.” – Inst 1, Leader
Q13	“To some extent in higher education innovation is limited by silos - if the research side is not talking to the clinical side and is not talking to the community engagement side, then we have barriers. We consciously wanted to break down those barriers to create innovation.” – Inst 3, Leader
Q14	“Any institution can become the one who knows what best practices looks like, but I think, both [community partner name] and I will say, based on our own impressions and experience coming from our Latino community and our African American community, that we do not have sense that academic institutions know us in our entirety and understand our either our histories or our subpopulation issues” – Inst 2, Community/Patient member
Q15	“What is [university name] idea of community? Is it these large government institutions or these really large community-based organizations, who have the capacity to push their agenda, or is it the “ground level,” as in everyday ordinary community folks who have a concern, who may even voice their concerns in community meetings, all of these places where the organizations that we trust to speak to us, where they’re supposedly gaining their influence from” – Inst 2, Community/Patient member

**Table 2.** Representative quotes for themes three and four

Theme 3: Institutional structures created power imbalances and community mistrust which were identified as key barriers to patient/community-engaged research	
Q16	“The gap I saw in looking, we have so many researchers and evidence-based work, but they never get scaled to change a community. What happens is people have a grant and they go out and say I have this and I’m going to give this to you, rather than “help us with this issue” … We do a lot of research and have a lot of grants, people having their own personal accolades, but most of that never gets to anyone [in the community.” – Inst 1, Leader
Q17	“I also would like to talk about the power structure. It has been really intimidating for me to approach researchers, as a patient, because these guys are brilliant. They’re brilliant scientists and they’ve got a whole bunch of letters after their name…the power dynamic, there’s just a huge gap there. It’s very hard to get involved, even if you find the time, even if you know you’re studying and trying to understand all of this. A lot of the researchers, maybe do not have the social skills to talk to patients they do not know that they’re very intimidating to patients but there’s not much of an effort to welcome patients to the table.” – Inst 1, Community/Patient member
Q18	“it’s a matter of having patients work with the researchers at the table in the design phase of studies or in the writing phase of a protocol. That is missing in large part from the patient researcher interactions at [University name]” – Inst 1, community/patient member
Q19	“In this [community engaged research] process, they need to stop having predetermined outcomes. What I mean by that is that if you ask me to be on a board or something for a grant, or something of that nature to flesh out, do not take your cookie cutter formula and stick it in there, and then say, okay, now fit it around this. Because then my input is of no value to you because you’ve already decided what you’re going to do, and that’s what I mean by having these predetermined processes. That also goes right directly back to what I was saying earlier, that you do not really hear me. The only thing that you’re wanting to do in this particular case or in those particular cases is check the box. “– Inst 1, Community/Patient member
**Theme 4: Institutional resources and investments are necessary for recruitment and retention of community-engaged researchers**	
Q20	“I think the barriers for equity are that the pool of candidates for faculty positions and leadership positions across different ethnicities is not as robust as others. So one of the things that this NCI mandate is looking at is diversifying leadership teams at cancer centers, and the leadership team should reflect diversity of the country itself. That will have a great impact I believe because they don’t right now.” – Inst 1, leader
Q21	“I would say …we have to continue grassroots advocacy for institutional structures and support, not a lot happens if at the highest level that is not something that is valued. I used to center on the article about collective impact… If we had an institutional mandate or some language to hang our work on. We’re all certified as community engaged faculty and that’s something that the institution values. It comes down to that high-level standard bearing.” – Inst 2, Investigator
Q22	“I came here from [university name], which as you all know, has extremely strong infrastructure that has been built over many years. When I came here, I felt very isolated in my role here and felt like I didn’t have people doing what I was doing. Then I found [faculty name] and some others and I thought, “Ok, these are my people. I want to work with them.” I think the junior faculty and the fellows are feeling that way too. We are trying to figure out if there is a way we can develop a forum of some kind where junior faculty can get feedback on some of their research projects or any ideas they have for research. Through this we also want to provide the mentorship they need from the few faculties that are coalescing here.” – Inst 2, leader
Q23	“All of our first-year medical students must take a yearlong community health course; each student is assigned to a group, we have sites for homeless, substance use… 13 community sites. These students led by the faculty will do a deep dive in the community. They do an environmental scan, research… They are engaged as partners in this year-long journey. At the end of the first semester, after all the research, the students determine with the community site in mind, what could be a community intervention that could help in a measurable way, outcome towards health equity. The faculty/staff/leadership at the community site partner with students and the professors to develop what the intervention looks like for the next semester intervention.” – Inst 3, Leader
Q24	“It’s not an Institutional barrier, It’s probably a structural barrier quite frankly -It’s always a money issue. Do we have enough money to go out? Can we at least give honoraria to these folks or a stipend or whatever, to show you value their time and work? I think that’s maybe the issue.” – Inst 3, Leader
Q25	“When you add community based participatory research they [university] have no idea what you’re talking about, so they assume you are working with a university, not a small community-based program and it’s such an uphill battle to work with our IRB and go up to, same problem with financial problems. Amount of times you have to go back and forth with finance people. Even if people in your department are good, they have to work with two to three levels of different financial people.” – Inst 2, Investigator

### Theme 1: Global pandemic and national events highlighting social injustices sparked a focus on health equity in academic health institutions

Many participants in the interviews and discussions highlighted the external pressures caused by the pandemic and the national events, suggesting that “Without a doubt everything that’s been going on in our country and around the globe, those events have brought people together. Sometimes feeling very vulnerable and threatened in a fashion where people come together to support one another, sometimes in a bit of anger or in a mode of ‘Oh this is a problem that we want to do our best to address.’” The leadership also noted that health and healthcare have been impacted by these events, with academic health institutions in a unique position to address these challenges, as noted by one leader in Q1. Leaders from all three institutions participating in this study highlighted the impact of the global pandemic and national injustices toward an important focus on health equity for academic health institutions. One leader noted that these events allowed for more strategic focus across the institution (Q2). Others mentioned leveraging these external events to undertake strategic prioritization within the academic health institutions, where one leader mentioned the pledge undertaken by the academic institution (Q3). Another leader (Inst 3) mentioned using the current events to, “do things differently, shift our model of operation in a way that allows us to do things differently and still have performance mechanisms in place.”

Quantitative data showed community and academic partners largely agreed, with most (65.8% of academic partners and 82.5% of community partners) respondents reporting that institutional statements on its mission, vision, and values demonstrated a commitment to health equity. However, among those community and academic partners that did believe that their institution held a commitment to promoting health equity, there was disagreement about the extent to which the institution was taking action to demonstrate that commitment. Among community partners, 79% believed that the institution was taking action toward health equity, but only 52% of academic partners agreed (Χ[2] = 6.466, *p* < 0.05).

Despite these strategic shifts, many leaders also mentioned that a focus on health disparities was not new for their work and for research, highlighting strategies such as telehealth were being incorporated prior to the pandemic (Q4). Many recognized, however, that the recent events had led to a broader understanding (Q5). Similarly, another institution participating in the study, suggested:

“We have been in the health disparity space since our existence, it’s part of our DNA. We believe that to some extent COVID-19 has allowed others to see what we have known for the last 40+ years. We knew very well COVID-19 would exacerbate what we know as health disparities, so we worked very quickly to mitigate those. Many people inside these communities have known it for years; now everyone is beginning to recognize it” - Inst 3, Leader

On the other hand, researchers highlighted a disconnect between leaders’ discussions of health equity and their observations of the institution. Survey data showed that only 36% of academics agreed that their institution was recognized for health equity research. Investigators also noted the recent emphasis on health equity needs to now be sustained through ensuring supportive structures are put in place for investigators and community members engaged in research. In one institution included in our study, a leader noted the importance of such a sustained focus (Q6). Investigators from other institutions included in this study also noted that the recent focus on health equity has created an important opportunity for ensuring institutional support, as noted by this investigator (Q7). Another investigator, also recognizing the recent shift in priorities especially in medicine, recognized that more conversations were considering community engagement in research (Q8).

### Theme 2: Including a focus on health equity did not translate to support for community-engaged research nor align with institutional reputation

Leaders across the participating health institutions mentioned several ongoing activities toward the goal of health equity. For example, a leader noted the overlaps with Diversity, Equity and Inclusion (DEI) efforts (Q9). Some mentioned that community-engaged approaches were just words that investigators used to recruit diverse populations (Q10). Another leader noted that,

“we’ve had perhaps a siloed understanding of what community engagement truly means. I think we need to make sure we have engaged and defined appropriately exactly the types of clinical guidance and partnership we need in so many other different domains.” – Inst 2, Leader

This recognition for sustained institutional partnerships also highlighted the need to operationalize and define what institutions meant, when they referred to the community. For example, leaders highlighted the multiple layers of community-engaged partners (Q11) and the need to differentiate between tokenism and authentic community engagement (Q12). Although it seemed that little effort was being placed on authentic community engagement, 68% of community members believe that the researchers they work with are comfortable developing an action plan to confront barriers to health equity that impact community members and patients.

Community partners also cautioned against partnering if there was no clarity from the academic health institutions in the purpose and intent of partnership, as described by a community member below:

“I’ve said before, academic institutions and research institutions use the community to further their goals, and very rarely is the community using the institution to assist them in their goals and I really think it’s important for [name of the institution] or any other institution to make it very clear how it is that the community and the institution can be working together and to make it really clear that it does not always have to come from the institution and often times it is the community that holds the solution and may need just a little bit of guidance and help to get there.. we may need some help in doing that research, to have the data, to back what it is they’re trying to do, because we know, when it comes to funding and things like that, people want hard data. So, that is where I see a major gap.” – Inst 2, Community/Patient member

Institutional reputation as leaders in basic and clinical sciences was mentioned across some of the participating academic health institutions, which made it difficult to prioritize populations sciences and community-engaged research. Quantitative findings showed that only 41% of academic partners thought their institution was recognized for its reputation in community- and patient-engaged research. One leader echoed this sentiment,

“[University name] is the place when it comes to mind for most people when you hear [University name] is fundamental discovery, basic science, hardcore, Nobel prizes, bench science. You don’t think public health, epi, clinical research, that’s not what you think/” – Inst 2, leader

Reputations of institutions combined with the foci for academic health institutions further perpetuated silos in partnering for community engagement (Q13). Only a third (32.6%) of academic partners agreed that institutional leaders support training and development of community-engaged scholars. Such perspectives of engaging with academic health institutions that did not truly understand the community surrounding them were shared strongly by the community and patient members participating in the assessment (Q14) and questioned the idea of how academic health institutions defined community (Q15).

### Theme 3: Institutional structures created power imbalances and community mistrust which were identified as key barriers to patient/community-engaged research

Many leaders and investigators from the participating sites mentioned critical barriers to supporting the ongoing community-engaged research, including a focus on research that does not directly address community priorities (Q16) or cannot be scaled in the community. Fewer than half of survey respondents (45%) agreed that institutional leaders support researchers to learn from community partners about the ways to address the environmental, social, and economic conditions that impact health. Community and patient members currently engaged in research projects mentioned that **“**providers and patients speak entirely different languages, and I observe it over and over…Providers have a very different agenda or a very different view of the world, and many patients, especially ones that are newly diagnosed, don’t understand the terminology and we don’t understand the treatments… it doesn’t matter which clinical group that you’re involved in, it seems like there’s just this incredible communication gap.” – Inst 1, Community/Patient member

While issues of trust were apparent from a historical perspective, some community and patient members also mentioned the power imbalances that arose in research projects (Q17). The power imbalances manifested in how investigators asked for community and patient input. Community members mentioned that interacting with patients and getting feedback on the patient experience was different from engaging patients in research. (Q18). Such engagement has to start at the design of the research project, and researchers should ensure that community/patient input is valued and incorporated throughout the research process (Q19).

### Theme 4: Institutional resources and investments are necessary for recruitment and ongoing support of community-engaged researchers

Across the institutions participating in this study, leaders noted the challenge of recruiting and retaining community-engaged researchers within their institutions (Q20). Other institutions participating in the study mentioned strategic investments in a “recruitment specialist,” who would

“go out and recruit the kinds of community based participatory research faculty that are going to help, look across the country, who are the people driving these agendas. [University name] has very clunky recruitment processes…but if you are an underrepresented minority or doing really impactful work in health disparities you can get a search waiver. We are trying to fast track some of these kinds of recruits that we think could really help change the complexion at [University name]” – Inst 2, Leader

In addition to recruitment, retaining existing faculty and supporting them in their community partnership was also mentioned by several leaders as an important support. However, among all survey respondents, 40% agreed that their institutions strongly support training and development of community-engaged scholars and 36% of academic respondents thought that institutional leaders supported researchers and staff to learn from community partners. Many investigators mentioned needing to advocate for themselves as valued members of the institution (Q21). In other institutions, the focus was on supporting investigators in an attempt to avoid the isolation that community-engaged researchers often experience in large academic institutions (Q22).

When queried about the specific types of institutional support to build capacity for that could strengthen P/CEnR, leaders, investigators, and community members suggested several strategies. In some cases, institutional leaders noted the importance of introducing authentic community partnership processes as a part of the medical school curriculum (Q23). Other suggestions included, “trying to find funding sources to help build infrastructure,” “ensuring that there were senior faculty with paid time [providing mentorship for community engagement in research],” and “recruiting a scientific editor to lead this subunit so we can help our faculty write and publish more.” Investigators also mentioned having to advocate for institutional support including time to engage with communities and sustain partnerships, ensuring that enough resources were provided to both the communities and investigators to avoid burning out. Only 27% of survey respondents agreed that the institution minimized barriers to participation of community partners in research.

Ensuring that community partners were adequately compensated was noted by many investigators and leaders as a priority. However, in some cases, ensuring that there was sufficient funding or fiscal departments not working in a timely manner for the compensation to reach the community was noted as a barrier (Q24, Q 25). Only 19% of academic partners agreed that their institution had necessary staffing resources to support CEnR. Specifically, only 33.3% of respondents agreed that institution made timely payments to community partners for participation in research and 26.6% agreed that institution made timely payments to community subcontractors.

### Theme 5. Despite challenges, participants were motivated to transform current paradigms of research and noted that accountability, communication, and training were key facilitators

Several leaders mentioned being motivated to incorporate health equity focus through community-engaged research, either due to the effects of the pandemic or because the institution wanted to establish themselves as a leader in this space (Q26). In some cases, leaders mentioned that the newer generation of students and post docs were demanding change to address community priorities, creating new pressures for the leadership (Q27). Community and patient members on the other hand thought that research that does not incorporate patient voice is “flawed,” as noted here by a participant:

“One of the issues is that when you are in research and you don’t have the patient’s voice - you have flawed research, because it’s from the perspective only from the researcher, and it’s not from the patient, which means that if you don’t have all the patient voices or the patients involved then you’re going to have the research being skewed one way. And so then I don’t think it’s effective research; any research project that doesn’t include community or patient is flawed.” – Inst 1, community/patient member

While noting that the academic health institutions supported broad research programs, “from ethics, humanities, population health measurements to health services research, outcomes research, to the most basic of sciences,” many participants mentioned that the programs were coming together by the importance of the healthcare equity focus in the past few years. Nonetheless, several leaders mentioned the need for a “more defined, systematic approach to the science of community engagement and the action and implementation of community engagement across every domain of our research enterprise.”

Many leaders and investigators brought up the key roles of institutional offices such as the Office of Community Outreach and Engagement that are typically established under varying names either in the Cancer Centers or the Clinical Translational Science Centers (Q28). Such offices that are typically supported by infrastructure grants could provide the resources for bringing community and patient partners to the table and supporting relationships, through a preexisting group of community-engaged investigators and need not be disease-specific. Investigators particularly thought these offices to be important (Q29), with community members noting the need to streamline the engagement with researchers (Q30).

Community partners also advocated for accountability from the academic partners, which was not limited only to the investigators engaging in community-based research but should be across the institution (Q 31). They challenged academic institutions to commit to the process, “by changing the makeup of the institution, stop inviting the same old people in. Invite some different people. Get comfortable with people who make you uncomfortable. That’s what shakes people up out of their status quo existence is when you get comfortable with being uncomfortable. And I don’t think that the institution as a whole is comfortable with that just yet, with different voices.” – Inst 2, Community/Patient member

### Joint display of themes and key quantitative findings

A joint display of the themes and supporting or opposing data from the quantitative assessments in provided in Table 4.

**Table 3.** Representative quotes for theme five

Theme 5. Despite challenges, participants were motivated to transform current paradigms of research and noted that accountability, communication, and training were key facilitators	
Q26	“The charge that the commission received was “tell us how we can become a national leader in this space,” that’s sort of the [Institution name] way, we like to be a national leader. But what the commission told the dean and the CEOs is “look, you are nowhere near ready to be a national leader in this space, and you need to lead locally before you can lead nationally.” So that was the message.” – Inst 2, Leader
Q27	“…if we are not translating discoveries into value propositions such that all those communities can realize the promise of science, right? No point in doing what we do. And so just the same way we think about how we’re educating and training this next generation for them to be able to help us eliminate disparities in care delivery and how we manifest and advocate for them in our public health programs. So, research plays an equal role and you do have to find it on the organizational chart sometimes for people to get that. It also matters in the investments that you make.” – Inst 3, leader
Q28	I think it requires more definition and more education of our faculty who don’t traditionally see this as a necessary component for their research. To make sure every investigator understands why it is so important for them to be working with the Office of Community Engagement for their human subjects research and to have a process that can support them just as we have a process for educating our junior faculty, to make sure it’s not restricted to junior faculty, but really making sure that people recognize that we’re not going to get the diversity of research participation, nor are we going to have our community fully understand the nature of clinical research unless we make this an integral requirement of every study.” – Inst 2, leader
Q29	“Having the OCOE is helpful because it’s hard to maintain relationships. You cannot just drop in, do your study, and leave - so having a more centralized place is really nice. I think by them supporting that center is a big sign that they do value it.” – Inst 1, Investigator
Q30	“You need to create a pathway through the Office of Community Engagement or an initiative that allows us to apply for a specific researcher, intern, a researcher to work on this specific kind of a data gathering, evaluation survey or project that we want, not what they need for furtherance of academic whatever they need to do, to have a place to go and apply, just like we do internships. That would be cool.” – Inst 2, Community/Patient member
Q31	“What policies are already present that sets accountability measures for [university name] as it relates to community research. I think that it’s not something that should be done department to department, but institution wide. If [university name] is saying we have this commitment to community engagement and research, then I think that that needs to be a part of their accountability measures, whatever that is. If there is not anything in place right now that speaks specifically to it and there is a way for the public to look and see what the measures are and are you really standing up to them, it’s something they need to do post haste because if it hasn’t even been addressed within the policies, then how serious are they? – Inst 2, Community/Patient member

**Table 4.** Joint display of qualitative themes and key quantitative findings

Qualitative themes	Key quantitative findings (n = 99)	Interpretations
**Theme 1:** Global pandemic and national events highlighting social injustices sparked a focus on health equity in academic health institutions	Approximately 65.8% of academic partners and 82.5% of community partners believe that the institutional mission, vision, and values statements demonstrate a commitment to health equity. Approximately 60% of academic partners and 76.7% of community partners believe that the institutional mission, vision, and values statements demonstrate a commitment to antiracism. Approximately 41% of academic partners and 59% of community partners believe that the institutional mission, vision, and values statements demonstrate a commitment to community-engaged research (Χ[2] = 5.519, *p* < 0.05).	Qualitative data highlighted the external influence of national events on promoting a focus on health equity, which were supported by high levels of agreement among patient/community respondents on the survey. Compared to the patient/community respondents, academic respondents showed lower levels of agreements on the institutional commitments to health equity, antiracism, and community-engaged research
**Theme 2:** Including a focus on health equity did not translate to support for community-engaged research nor align with institutional reputation	52% of academic partners and 79% of community partners believe that the institution is taking action on the commitment to health equity (Χ[2] = 6.466, *p* < 0.05) 58.3% of academic partners and 87% of community partners believe that the institution is taking action on the commitment to antiracism (X[2] = 3.48; *p* = 0.06). 72.2% of academic partners and 82.8% of community partners believe that the institution is taking action on the commitment to community-engaged research. 41.9% of academic partners and 72.1% of community partners believe that the institution is recognized for its reputation in community- and patient-engaged research (X[2] = 8.07; *p* = 0.02). 37.2% of academic partners and 71.8% of community partners believe that the institution is recognized for its reputation for health equity (X[2] = 9.93; *p* = 0.007).	Much of the qualitative data came from the leadership and the academics in supporting a lack of focus on community-engaged research at the academic health institution, as was supported in the quantitative data. As reported earlier, patient/community members that participated in the survey had much more positive perceptions of actions taken by the institutions with significant differences noted between them and the academics. Similarly, there were significant differences among how academics and patient/community members taking the survey perceived institutional reputation.
**Theme 3:** Institutional structures created power imbalances and community mistrust which were identified as key barriers to patient/community-engaged research	71.8% of academic partners and 74.4% of community partners, believe that the researchers can reflect and identify systems of power that influence treatment of patient/community members 41.7% of academic partners and 80% of community partners believe that institutional leaders support researchers to learn from community partners about the ways to address the environmental, social, and economic conditions that impact health (X[2] = 9.95; *p* = 0.007). 86.2% of academic partners and 82.8% of community partners believe that researchers and staff are willing to change how we conduct research in response to community and patient advocate feedback 27.8% of academic partners and 64.1% of community partners believed that the institution offered education for patient and community partners on research processes (e.g., grant writing, data analysis, disseminating results, etc.) (X[2] = 10.23; *p* = 0.006). 12.1% of academic partners and 70.0% of community partners believe that the institution has policies that require patient and community partners to review grant applications for community benefit (X[2] = 22.26; *p* < 0.001). 11.4% of academic partners and 65.6% of community partners believe that community members and patients are involved in strategic planning for the organization at the top institutional leadership level (X2 = 21.00; *p* < 0.001).	Although qualitative data highlighted power imbalances and mistrust as key barriers for patient/community members, many community survey respondents reported more positive perceptions of supportive institutional structures, likely due to their own involvement in community-engaged research. Survey findings highlighted, several points of divergence noted among community and academic survey respondents as they related to the policies and the resources offered by the institutions, most likely due to limited or lack of knowledge about the institutional policies among community respondents.
**Theme 4:** Institutional resources and investments are necessary for recruitment and ongoing support of community-engaged researchers	Only 19% of academic partners and 45% of community partners agree that their institution has the necessary staffing resources to support community-engaged research (Χ[2] = 8.157, *p* = 0.017) 23.5% of academic partners and 74.2% of community partners agreed that the institution has IRB policies and practices that support patient and community-engaged research projects (Χ[2] = 17.195, *p* < 0.001) 17.6% of academic partners and 51.5% of community partners agreed that the institution has funding strategies to mobilize community partners to research health inequities (Χ[2] = 13.010, *p* = 0.002) 19.4% of academic partners and 65.6% of community partners agreed that the institution has written standards that provide expectations for staff and faculty for conducting patient and community-engaged research (Χ[2] = 14.692, *p* = 0.001) 16.7% of academic partners and 62.5% of community partners agreed that the institution includes community and patient engagement products (e.g., policy briefs, reports to community organizations or government agencies, etc.) into tenure and promotion guidelines for faculty (Χ[2] = 12.526, *p* = 0.002) 36.8% of academic partners and 67.7% of community partners agreed that institutional leadership strongly supports the training and development of community- and patient-engaged scholars (Χ[2] = 10.673, *p* = 0.005).	Supporting the qualitative data, there were several points of divergence noted among academic and community survey respondents, most like due to a lack of or limited knowledge about institutional policies among community survey respondents. Academic respondents reported low levels of agreement for institutional resources like staffing, IRB policies, and funding for P/CEnR. Similar to the qualitative data, many academic respondents noted low levels of agreement for policies that could support the promotion of community-engaged scholars in the institution.
**Theme 5:** Despite challenges, participants were motivated to transform current paradigms of research and noted that accountability, communication, and training were key facilitators	86.2% of academic partners and 90.3% of community partners agreed that researchers mobilized partnerships to address social determinants impacting health outcomes 81.5% of academic partners and 85.7% of community partners agreed that researchers and staff regularly evaluate how partnership is going and what can be done to improve. 70.7% of academic partners and 76.7% of community partners agreed that institutional leadership encourages researchers and staff to engage in health equity research. 89.7% of academic partners and 83.9% of community partners agreed that the office of community engagement contributes to advocating for policies that address conditions that affect health inequities 96.8% of academic partners and 83.9% of community partners agreed that the office of community engagement contributes to new insights, innovative solutions, and the evidence base to address health inequities and community conditions that influence health	Similar to the qualitative data, both academic and community respondents in the survey reported high levels of agreement with institutional priorities to support P/CEnR through mobilization of partnerships, reflections, and encouragement to engage in health equity research. Survey respondents also highlighted the role of the office of community engagement, when queried specifically about their role in advocating for policies, and contributing to new insights for addressing inequities, which was also supported in the qualitative data.

## Discussion

This Engage for Equity (E2) PLUS mixed-methods study of contextual facilitators and barriers has validated our own understanding from our previous Engage for Equity (E2) research [22], of the limitations of single investigator-led research to create sustainable P/CEnR infrastructures within academic health institutions. Our theoretical saturation with the 42 qualitative respondents and 99 survey respondents confirmed the importance of understanding the contextual factors that facilitate or are barriers to institutional transformation. This recognition is important for future practice and research as academic health institutions seek to create contextually based strategies for strengthening patient and community-engaged research infrastructures. Mixed-methods analyses uncovered contextual determinants that also mirror a growing literature articulating the administrative and financial challenges to developing effective policies and practices that demonstrate support for engaged research [8,11,36]. This E2PLUS study however added a theoretical framework-driven understanding of new dimensions that were revealed through multiple (i.e., leaders, researchers, and community members and patients) perspectives. For example, the role of external context, in particular the role of COVID in shaping a recommitment to health equity and racial justice, yet the challenge remained in translating this stated commitment to health equity. Although the external context catalyzed a focus on health equity, there are gaps in translating that momentum to P/CEnR. As supported in our previous research community/patient–academic partnerships are unsustainable if the academic health center does not provide support through policies, practices for both fiscal and administrative support toward engaged research [24].

What was of particular interest was the divergence in the quantitative and qualitative findings among community/patient advocates and academic top leaders and investigators, with community members drawing from their historical observations of lack of accountability of the institution or of NIH to the community. Clearly articulated were imbalances in power for research decision-making, and a lack of resources for sustained patient/community involvement. This study also highlighted the nuances of engaging patients or caregivers with lived experiences of health conditions in research, who may have the goal for advocating for themselves or their patient partner, which need additional support and engagement [37,38]. In some cases, community partners had favorable views of P/CenR, likely because their specific academic partners may have attempted to reduce the barriers they faced, highlighted by the high trust reported by community partners in their academic partners. Building on the lessons learned from this work, we hope to further highlight the different approaches that might be necessary to engage patients and community members.

Despite the barriers and some of the differences, there surfaced a theme of commitments to transform the research enterprise, with specific strategies of communication, support for investigators, community accountability, and need for more resources identified. An important finding was the role of institutional offices, such as Offices of Community Outreach and Engagements in Cancer Centers [39], Community Engagement Cores in Clinical Translational Science Centers [40], Prevention Research Centers, and federally funded centers, with community partners seeing them as having more influence, than the investigators who had more insider knowledge and could articulate the need for greater resources and top leaders support. These offices were perceived to be strategically positioned to build support for community engagement by bringing together representative from these offices across the academic health institution since many of these offices existed within an institution. The challenge, however, remains in supporting the individuals (i.e., staff, researchers, and leadership) in these strategic offices through structural changes at the institutional level to avoid tokenism[41] and ensure that research incorporates the voices of patients and community members [42]. These data and co-interpretation of these data provided opportunities for initial attempts at coordinating activities across the academic health institution. Future work may need a strong emphasis on ensuring consistent communications and coordination, and resources between these offices to ensure a united front for academic institutions engaging with community partners.

### Strengths and limitations

Building on extensive experience in evaluating and supporting P/CenR, this study was one of the first attempts at exploring and understanding how to address the institutional support for community/patient engagement in research. As a starting point, this study mostly engaged investigators and community/patient members that were already participating in research studies. Future research should also examine these perspectives from individuals that may not be actively participating in engaged research and may or may not share similar perspectives. In analyzing the data, we recognized our limitation of not having a clear understanding of the context in which the community-based organizations operate, or the relational context between the community-based organizations and institutions, which may have an important influence on outcomes for P/CEnR. Future research may incorporate both institutional perspectives, from the academic health centers and community-based organizations. Finally, from a methodological perspective, we triangulated across methods (i.e., quantitative vs qualitative) and across the respondents and may not have adequately represented each perspective included in this study. This study was limited to three institutions that were willing and ready to partner on validating the Engage for Equity methods and metrics on an institutional level. Future work to explore institutional assessments must consider and address differences across respondents, methods, and institutions.

## Conclusions

Requirements from National Institutes of Health, particularly National Clinical and Translational Science Institute and the National Cancer Institute, have incorporated an institutional focus on supporting and promoting community/patient–academic partnerships, through their community engagement offices and centers. Such requirements provide a critical opportunity to leverage institutional structures and processes to support community/patient-engaged research. Study findings provide an in-depth and theory-guided assessment of institutional context that can provide several strategies and mechanisms by which institutions could address the hurdles to promote P/CEnR, further highlighting the importance of engaging existing institutional representatives in the CBPR approach to design sustainable solutions. They highlight a novel focus on academic health institutions as important contextual influences and provide important targets for interventions to improving supportive policies and practices toward equity-based P/CEnR.
